# High Throughput Mutagenesis for Identification of Residues Regulating Human Prostacyclin (hIP) Receptor Expression and Function

**DOI:** 10.1371/journal.pone.0097973

**Published:** 2014-06-02

**Authors:** Anke Bill, Elizabeth M. Rosethorne, Toby C. Kent, Lindsay Fawcett, Lynn Burchell, Michiel T. van Diepen, Anthony Marelli, Sergey Batalov, Loren Miraglia, Anthony P. Orth, Nicole A. Renaud, Steven J. Charlton, Martin Gosling, L. Alex Gaither, Paul J. Groot-Kormelink

**Affiliations:** 1 Developmental and Molecular Pathways, Novartis Institutes for Biomedical Research, Cambridge, Massachusetts, United States of America; 2 Respiratory Disease Area, Novartis Institutes for Biomedical Research, Horsham, United Kingdom; 3 Infectious Diseases, Genomics Institute of the Novartis Research Foundation, San Diego, California, United States of America; 4 Musculoskeletal Disease Area, Novartis Institutes for Biomedical Research, Basel, Switzerland; Cleveland Clinic Lerner Research Institute, United States of America

## Abstract

The human prostacyclin receptor (hIP receptor) is a seven-transmembrane G protein-coupled receptor (GPCR) that plays a critical role in vascular smooth muscle relaxation and platelet aggregation. hIP receptor dysfunction has been implicated in numerous cardiovascular abnormalities, including myocardial infarction, hypertension, thrombosis and atherosclerosis. Genomic sequencing has discovered several genetic variations in the PTGIR gene coding for hIP receptor, however, its structure-function relationship has not been sufficiently explored. Here we set out to investigate the applicability of high throughput random mutagenesis to study the structure-function relationship of hIP receptor. While chemical mutagenesis was not suitable to generate a mutagenesis library with sufficient coverage, our data demonstrate error-prone PCR (epPCR) mediated mutagenesis as a valuable method for the unbiased screening of residues regulating hIP receptor function and expression. Here we describe the generation and functional characterization of an epPCR derived mutagenesis library compromising >4000 mutants of the hIP receptor. We introduce next generation sequencing as a useful tool to validate the quality of mutagenesis libraries by providing information about the coverage, mutation rate and mutational bias. We identified 18 mutants of the hIP receptor that were expressed at the cell surface, but demonstrated impaired receptor function. A total of 38 non-synonymous mutations were identified within the coding region of the hIP receptor, mapping to 36 distinct residues, including several mutations previously reported to affect the signaling of the hIP receptor. Thus, our data demonstrates epPCR mediated random mutagenesis as a valuable and practical method to study the structure-function relationship of GPCRs.

## Introduction

The human prostacyclin (PGI_2_) receptor (hIP receptor, International Union Pharmacology nomenclature) is a seven transmembrane (TM) G protein-coupled receptor (GPCR) [1]. The 386 amino acid protein is encoded by the human prostacyclin receptor gene (PTGIR) and is composed of a short N-terminal tail, seven TM-spanning alpha-helical domains, three intra- and three extracellular loops and a long c-terminal tail including two palmitoylation sites. The receptor is further stabilized by two disulfide bonds [2], [3]. The hIP receptor is activated by binding of prostacyclin which leads to the activation of membrane-bound adenylyl cyclase, subsequent formation of the second messenger cyclic adenosine monophosphate (cAMP) and activation of various cellular responses [1]. The hIP receptor is ubiquitously expressed in the body, but shows predominant expression in the cardiovascular system, especially on platelets and vascular smooth muscle cells where it plays a key role in vascular smooth muscle relaxation and inhibition of platelet aggregation. Recent studies have revealed a cardioprotective role of the IP receptor and hIP receptor dysfunction has been implicated in numerous cardiovascular abnormalities, including myocardial infarction, hypertension, thrombosis and atherosclerosis [4]–[9]. Furthermore, genetic variants in the hIP receptor resulting in deficits of hIP receptor function have been correlated with increased disease severity in patients with coronary artery disease [10], [11]. Thus, it is of paramount significance to understand the mechanisms and structural requirements underlying hIP receptor function. Despite significant progress in our understanding of GPCRs in general and multiple studies using site-directed mutagenesis to identify residues critical for agonist binding and hIP receptor activity [9], the details of hIP receptor structure-function remain largely unknown.

Here we set out to explore the relationship between hIP receptor structure and function using an unbiased, high-throughput, random mutagenesis approach. We compared the applicability of chemical mutagenesis and error-prone PCR mediated mutagenesis to create a library covering the complete coding region of the hIP receptor and analyzed the quality of our library by next generation sequencing (NGS). Furthermore, we report the functional characterization of >4000 hIP receptor mutants and the identification of 18 mutants of the hIP receptor that maintained expression but demonstrated a full or partial reduction in receptor activity. Our results highlight the advantages of random high throughput mutagenesis to gain insights in the structure-function requirements of hIP receptor, as compared to biased approaches using site-directed mutagenesis.

## Experimental Procedures

### Hydroxyl amine mutagenesis

Hydroxylamine mutagenesis, which reacts with pyrimidine nucleotides to generate cytosine to thymidine transitions, was adapted from a previous published protocol [12]. 5 ug HA-tagged hPTGIR in pcDNA5 was incubated with 250 µl 1 M hydroxylamine hydrochloride (Sigma Aldrich), pH 7 at 70°C for 32 to 128 min. Mutagenised plasmids were then isolated and washed at least 3 times using the QiaQuick PCR purification kit (Qiagen), and finally eluted with 50 µL EB buffer from the kit and diluted with 50 µL sterile water. 1 µL of purified mutated plasmid was transformed into 100 µL Invitrogen Library Efficiency DH5α competent *E. coli* (Invitrogen). Transformed cells were then plated onto Luria Broth (LB)-agar media supplemented with ampicillin and grown O/N at 37°C. Individual bacterial colonies were picked and grown O/N at 37°C, DNA was isolated, the hPTGIR insert sequenced and the mutation rate per mutant determined (Table 1).

**Table 1:** Colony number and mutation rate obtained by hydroxyl amine mediated mutagenesis.

Incubation	# colonies	# colonies	# colonies with indicated number of mutations				mutation
time	obtained	sequenced	0 mut	1 mut	2 mut	>2 mut	rate
128′	16	16	10	6	0	0	0.38 mut/kb
64′	∼800	62	53	7	1	1	0.15 mut/kb
32′	∼1500	56	53	3	0	0	0.05 mut/kb
16′	∼2000	30	30	0	0	0	-
12′	∼3000	44	44	0	0	0	-
8′	>10,000	30	30	0	0	0	-
4′	>10,000	40	40	0	0	0	-
2′	>10,000	30	30	0	0	0	-
1′	>10,000	30	30	0	0	0	-
0′	>10,000	30	30	0	0	0	-

### Error-prone PCR mediated mutagenesis

Error-prone PCR (epPCR) mediated mutagenesis was performed using the GeneMorph II Random Mutagenesis kit (Stratagene). Briefly, 500–1000 ng of plasmid DNA (pcDNA5-HA-hPTGIR) was mutagenized by PCR according to the manufacturer's protocol using the following primers: IPR-for: 5′- CGACTCACTATAGGGAGACCCAAGCTGG -3′, IPR-rev: 5′- CAACTAGAAGGCACAGTCGAGGCTGATC -3′. PCR products were separated by gel electrophoresis and purified using a gel-extraction kit (Qiagen). After restriction enzyme digest (HindIII and XhoI), the DNA was purified with a PCR purification kit (Qiagen) and cloned into the parental vector (pcDNA5-HA). Mutagenized DNA was transformed into *E.coli* DH5α Max Efficiency (Invitrogen). For initial studies to determine the mutation rate, between 100–200 single colonies were handpicked. For the large mutant library prep around 4000 individual colonies were picked with a Mega-Pix robot (Genetix). In both cases, colonies were transferred to 96-deep well blocks containing 1.2 mL of terrific broth (TB)/Carbenicillin/Tylosin, and grown at 37°C O/N in the HiGro incubator (Gene Machines, Inc.). The next day glycerol stocks were made of the whole library by transferring 100 µl of the bacterial culture into 70 µL of 50% glycerol/50% TB solution. The glycerol stocks were stored at −80°C for future amplification of the library. DNA purification of mutant clones from the remaining 96-deep well bacterial cultures was performed by a RoboSeq 4204 prep robot (MWG Biotech) with the NucleoSpin Robot-96 Plasmid Miniprep kit (Macherey-Nagel). Purified plasmids in 96-well microtiter plates were quantified with a PowerWave XS spectrometer (Bio-Tek) and normalized to a final concentration of 40 ng/µL with a TheOnyx (MWG Biotech) liquid-handling robot. The 96-well plates containing normalized mutant DNA were compressed into 384-deep well barcoded bioassay plates (Greiner Bio-One) by transferring 62.5 µL of mutant plasmid with a MiniTrak robot (Perkin Elmer) into assigned 384-well plate coordinates. In total, twelve unique 384-well plates (approx. 4,000 mutants) containing an individual clone within each well were generated. All plates were stored at −20°C.

### Next generation sequencing

All library constructs were pooled and the genomic library was prepared and sequenced by Axeq Technologies on an Illumina HiSeq 2000. The 101 bp paired-end reads were aligned to the wild-type-sequence of human PTGIR using BWA (v0.7.4) with the parameters -n 2 -t 12 (pmid 19451168). Alignments were converted to bam (pmid: 19505943) and only high quality nucleotides were counted at each position using the bamutils basecalls function (pmid 23314324; bamutils basecall -ref wildtype.fa -qual 40 epPCR.bam). Mutations were called against the reference sequence and mutation rates calculated with R and the plyr package (R Development Core Team (2008). R: A language and environment for statistical computing. R Foundation for Statistical Computing, Vienna, Austria. ISBN 3-900051-07-0, URL http://www.R-project.org; [13]). Non-biased assessment of codon usage was calculated by dividing the total mutations across the coding sequence by the potential mutations. All potential codons were then determined based on the three alterative bases at each location, assuming one mutation per codon. The data has been deposited in the NIH SRA database (BioProject: PRJNA242280).

### Cell culture and transfection

HEK293 GloSensor-20F cAMP cells (Promega) were maintained in DMEM supplemented with foetal bovine serum (10% v/v) and hygromycin (200 µg/mL) at 37°C, 5% CO_2_. For experiments, cells were harvested using trypsin/EDTA buffer and seeded on to multiwell plates in CO_2_-independent medium containing foetal bovine serum (10% v/v). Reverse-transfection of cells with the mutant library was performed in 384-well black, clear bottom plates using FuGENE6 (Promega) according to manufacturer's instructions. Briefly, 1 µL of cDNA library or wild-type hIP receptor (40–100 ng) was seeded into the 384-well assay plate using the Mosquito nanoliter dispenser. FuGENE6 was diluted in OptiMEM to give a 1∶3 DNA:FuGENE ratio (for 40 ng cDNA, 0.12 µl FuGENE6 per well in 4 µl OptiMEM/384 well). 4 µl OptiMEM/FuGENE mixture was then added to cDNA in 384 well plate, and incubated at room temperature for 30 minutes, with gentle shaking. 15 µl of cells (0.67×106 cells/mL; 10,000 cells/well) was then added to the DNA/OptiMEM/FuGENE mixture. Cells were then incubated overnight at 37°C, 5% CO_2_.

### GloSensor cAMP assay

Kinetic analysis of cAMP accumulation in cells expressing hIP receptor was conducted using transiently transfected cells. 10 µL of 12% GloSensor reagent (4% [final]) in CO_2_-independent medium containing FBS (10% v/v) was added to the cells and the plate was incubated for 2 hour in the dark at room temperature. Luminescence (analogue mode, sensitivity 2) was then read on the FDSS7000 every 1.5 sec (integration time of 1.2 sec), for a total of 1000 samples. 5 µL of 7x MRE-269 [4-[(5,6-Diphenylpyrazin-2-yl)(isopropyl)amino]butoxy] acetic acid (synthesized in house) was transferred to the 384-well cell plate using the FDSS7000, after the first 5 samples. Hits were characterized as mutants that resulted in <25% of the response to MRE-269 in cells transfected with the wild-type hIP receptor.

### Receptor expression

The above assay was then repeated with expression analysis. After transfection of selected mutant hIP and wild type receptors, cells were incubated in CO_2_-independent medium containing FBS (10% v/v); GloSensor reagent (4% v/v); Hoechst (1 µM); Alexa Flour® -647 conjugated anti-HA antibody (1∶500). Receptor expression was analyzed after 30 min on the InCell2000. Nuclei were visualized using DAPI settings (0.05 s exp), HA-tagged receptor using Cy5 settings (1.5 sec exp) and cells were visualized using bright field settings (0.05 s exp). The expression of mutant receptor was analyzed using InCell Developer Toolbox 1.9.1 software. Cells were detected using Object Segmentation (kernel size  = 57, Sensitivity  = 55), followed by Fill Holes and Sieve (Binary) based on size (>10 µm^2^). Mean and Sum of the fluorescence detected was taken for each well as an estimate of the relative expression levels for each mutant receptor. Cells were then incubated further at room temperature (2 hour in total) and activity assessed as above.

## Results and Discussion

### Chemical mutagenesis

Mutations in the hIP receptor and impaired receptor function have been correlated with cardiovascular disease [10], [11], however little is known about the structure-function relationship of the receptor. The hIP receptor has been intensively studied by site-directed mutagenesis, leading to the identification of critical residues for receptor activation and ligand binding [9], [11], [14]–[17]. However, because of the labor-intensive nature of this approach, these studies were focused on a limited number of residues that were selected based on homology with other receptors or molecular modeling approaches and are therefore highly biased. In order to identify residues important for hIP receptor function we set out to generate an unbiased library of hIP receptor mutants, covering the complete coding region of the receptor, for subsequent functional characterization.

In contrast to site-directed mutagenesis, random mutagenesis does not require the *a priori* selection of particular residues but rather allows for the generation and testing of thousands of amino acid substitutions in an unbiased way. However, depending on the mutagenesis method used, different degrees of bias regarding the type of substitution made can be achieved.

Chemical mutagenesis is the most convenient method to mutagenize a plasmid, since it does not involve any recombinant manipulation or sub-cloning of the parental DNA, *i.e*. generally the parental plasmid is incubated directly with one of several chemicals and can be used directly for transformation after a simple purification step [18]–[21]. Chemical mutagenesis can be especially useful if low mutation rates are required or sub-cloning of the targeted region is not possible. An obvious disadvantage is the lack of specificity for a specific region and the limitation of possible base pair changes, resulting in a highly biased library.

We set out to test the applicability of chemical mutagenesis to generate a mutant library for the hIP receptor to gain additional, unbiased insights into the relationship between the structure and function of this receptor. For this, we incubated the parental plasmid coding for the hIP receptor with 1 M hydroxyl amine for 32 min–128 min. Treatment of double stranded DNA with Hydroxyl amine is known to result in C->T and G->A transitions thus allowing for only a limited fraction of possible substitutions [21]. After a purification step, the DNA was transformed in *E.coli*. As shown in Table 1 the number of resulting colonies was inversely correlated to the time used for incubation with hydroxyl amine. Longer incubation times are expected to result in higher mutation rates, increasing the probability of mutations in regions of the vector coding for the ampicillin resistance used for selection, resulting in a loss of resistance. To determine the mutation rate in the coding region of the hIP receptor, a representative number of colonies were subsequently analyzed by Sanger sequencing. The observed rate of mutations in the coding region of the hIP receptor was very low, with an observed maximum of approximately one mutation every 2.6 kb (Table 1). Notably, the number of colonies obtained under these conditions would not suffice to generate a mutagenesis library of sufficient size to cover the coding region of hIP receptor. These results demonstrate the suitability of hydroxyl amine mediated mutagenesis for the generation of libraries with low mutations rates. For the purpose of our study, however, the observed mutation rate was not sufficient.

### Error-prone PCR mediated mutagenesis

Next we explored the feasibility of epPCR mediated mutagenesis to generate a mutant library for the hIP receptor. epPCR is fast and facile, and the most commonly used method to create random mutagenized libraries [22]. It is used to introduce random mutations during PCR by compromising the fidelity of DNA polymerase, resulting in the incorporation of incorrect nucleotides during the PCR reaction and yielding randomly mutated products [23]. The fidelity of the DNA polymerase and therefore the error rate can be reduced by altering the reaction conditions for the PCR, *i.e*. ion concentrations, amount of starting material, number of cycles, or by using special variants of DNA polymerases with known deficits in 3′-5′ exonuclease activity [22], [24]–[26]. After the epPCR, the library of mutated DNA sequences is cloned in a suitable plasmid, making an additional purification and ligation step necessary, but on the other hand ensuring that no mutations are present outside the region of interest in the expression plasmid used. A disadvantage of epPCR mutagenesis lies in the mutational preference of the low-fidelity DNA polymerases used to introduce the mutations, favoring substitution of certain nucleotides over others resulting in a biased library [27], [28]. One approach to reduce the bias of the library and to increase diversity is to combine two DNA polymerases with opposite mutational spectra [29]. This approach has been proven to significantly reduce the bias in the library and is used by multiple commercially available mutagenesis kits. We used the commercially available Genemorph II kit which uses a mixture of two polymerases, *Taq* and *Mutazymell*, with opposite mutational spectra to reduce mutation bias.

**Figure 1:**
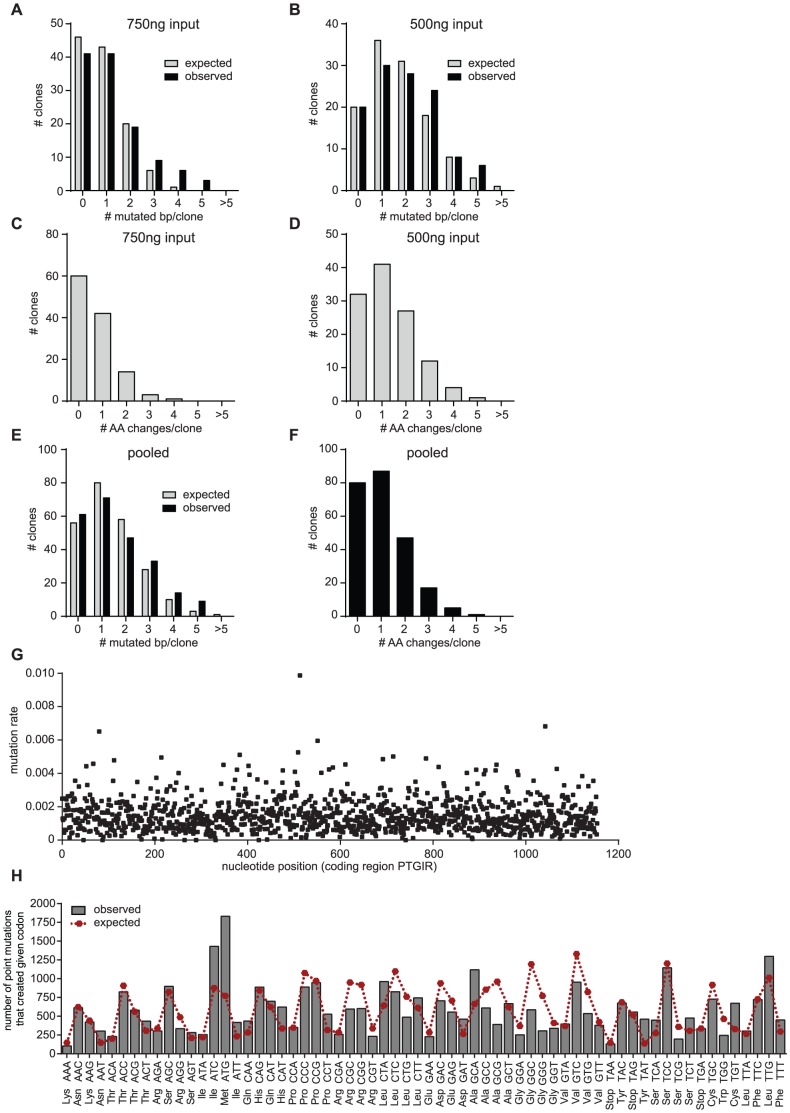
Error-prone PCR mediated mutagenesis of hIP receptor. (A, B) Number and distribution of mutations per clone after error-prone PCR mediated mutagenesis of hIP receptor using 750 ng (A) or 500 ng (B) parental PTGIR plasmid as input. Grey bars represent the expected mutational spectrum at a mutation rate of 0.8 mut/kb (750 ng) or 1.5 mut/kb (500 ng), respectively. Black bars represent the experimentally observed number of mutations after sequencing a small subset of clones. (C, D) Expected number and distribution of amino acid changes per clone at a mutation rate of 0.8 mut/kb (750 ng, C) or 1.5 mut/kb (500 ng, D) (E) Expected and observed number and distribution of mutations after pooling both libraries (500+750 ng) depicted as described in (A,B). (F) Expected number and distribution of amino acid changes per clone in our library with an estimated mutation rate of 1.3 mut/kb. (G) Scatter blot showing the mutation rate for each nucleotide position of the coding region of PTGIR as determined by next generation sequencing. The overall mutation rate was 1.48 mutations/kb. (H) Comparison of the observed codon changes in our library (bars) with the expected codon changes (considering an unbiased mutation rate and one nucleotide substitution per codon, red symbol).

The estimated mutation frequency can be described as a product of the DNA polymerase error rate and the number of amplifications [30]. The more often a target is replicated, the more errors are accumulated. Therefore the initial DNA amount can be used to adjust the mutation frequency. We performed three independent epPCR reactions with varying template DNA concentrations, sub-cloned the PCR fragments in a plasmid including an HA-tag and analyzed the mutation rates by sequencing a small subset of the resulting colonies after transformation in *E.coli*. As expected, the highest input of starting material (1 ug) resulted in a low mutation rate of approximately 0.5 mutations per kb, the lower DNA input of 750 ng and 500 ng yielded significantly higher mutation rates of approximately 0.8 and 1.5 mutations/kb, respectively (Table 2, Figure 1 A, B).

**Table 2:** Colony number and mutation rate obtained by error-prone PCR mediated mutagenesis.

DNA	# colonies	# colonies with indicated number of mutations							mutation
input	sequenced	0 mut	1 mut	2 mut	3 mut	4 mut	5 mut	>5 mut	rate
500 ng	116	20	30	28	24	8	6	0	1.5 mut/kb
750 ng	120	41	41	19	9	6	3	0	0.8 mut/kb
pooled	236	61	71	47	33	14	9	0	1.3 mut/kb

### Simulation of optimal mutation rate

Low mutation rates lead to libraries with many functional sequences (*i.e*. wild-type sequences and mutants with only a single amino acid change), but small numbers of total mutations and therefore overall low coverage (*i.e*. frequency with which a particular amino acid has been mutated in the library). High mutation rates enrich libraries for sequences with high numbers of mutations therefore leading to less functional sequences and a good coverage. However, the accumulation of mutations leads to a decrease in the unique mutation rate (*i.e*. how often a particular amino acid has been changed without any other mutations occurring in the same plasmid) [31]. Thus, one has to find the optimal mutation rate that balances overall mutation rate (coverage), functional retention and uniqueness. Low mutation rates with maximal unique mutation rate are optimal, but require a larger number of mutants (library size) to guarantee sufficient coverage. The distribution of the number of mutations per construct is expected to be a binomial distribution, and for the ease of downstream analysis of the ensemble of mutants we aimed to maximize the number of single amino acid mutations and minimize multiple and null mutations (*i.e*. the mode of the distribution would be at 1). The penetrance of DNA mutations into amino acid mutations is incomplete, and by simple *in silico* simulation for all positions in a representative set of genes, we determined the penetrance correction coefficient to be 0.75 (for the target genes in the study, the coefficient was between 0.72 and 0.77). We have prepared a simple worksheet that allowed us to simulate the expected number and distribution of amino acids in our library as a function of the mutation rate (see Table S1). Using this algorithm, we simulated the expected number and distribution of amino acids in our library as a function of the mutation rate and compared it to the distribution obtained by sequencing a small number of mutated colonies (Figure 1C, D). As can be seen in Table 3, a mutation rate of 1.1 mutations/kb is expected to produce the most single amino acid changes, but at the same time retaining 35% wild-type (non-mutated or silence nucleotide mutations) sequences. The lowest fraction of wild-type sequences in combination with high fractions of single and dual amino acid changes can be obtained with a mutation rate of 1.5 mutations/kb. Therefore, we pooled our two epPCR reactions, resulting in a library of >4000 hIP receptor constructs with a mutation rate of approximately 1.3 mutations/kb (Figure 1E, F).

**Table 3:** Estimated distribution of amino acid (AA) changes relative to the mutation rate (library size: 4000 plasmids).

mutation	Estimated number of plasmids with indicated number of amino acid (AA) changes			
rate	0 AA	1 AA	2 AA	>2 AA
0.5 mut/kb	2494	1179	278	49
0.6 mut/kb	2269	1287	365	80
0.7 mut/kb	2064	1366	452	117
0.8 mut/kb	1878	1420	537	164
0.9 mut/kb	1708	1454	618	219
1.0 mut/kb	1554	1470	694	283
1.1 mut/kb	1414	1471	765	350
1.2 mut/kb	1286	1460	828	427
1.3 mut/kb	1170	1439	884	507
1.4 mut/kb	1065	1410	933	592
1.5 mut/kb	969	1374	974	682
1.6 mut/kb	881	1334	1009	777
1.7 mut/kb	802	1289	1036	873
1.8 mut/kb	729	1242	1057	971
1.9 mut/kb	663	1193	1071	1073
2.0 mut/kb	603	1142	1080	1173
2.1 mut/kb	549	1091	1083	1276
2.2 mut/kb	499	1040	1082	1379
2.3 mut/kb	454	989	1076	1481
2.4 mut/kb	413	939	1066	1582
2.5 mut/kb	376	890	1052	1683

### Analysis of mutagenesis libraries by next generation sequencing provides valuable information about coverage, mutation rate and mutational bias

Sanger sequencing is a rapid and cost-effective method to determine the mutation rate of a subset of clones which can be used to predict the expected mutation rate of the library. Due to limitations in throughput, scalability and coverage range, sequencing efforts are typically focused on a subset of clones only. However, sequencing of only a subset of clones does not provide reliable information about the mutational coverage of the targeted region or the expected mutational bias. NGS has made it possible to process multiple sequencing reactions in parallel, enhancing throughput and allowing for better coverage and resolution. To analyze the quality of our mutagenesis library and to obtain more reliable information about coverage and mutation rate, we sequenced our complete mutagenesis library for the hIP receptor using NGS. The pooled library was sequenced on an Illumina HiSeq 2000 (Axeq Technologies); the 101 bp paired-end reads were aligned to the wildtype-sequence of hIP receptor and the mutations were assessed (Table 4). The average coverage for the coding region of hIP receptor was ∼21000 reads. The data revealed a homogenous coverage of the complete coding region of hIP receptor with only a few outliers (Figure 1G). The overall mutation rate was 1.48 mutations/kb, very similar to our predicted mutation rate of 1.3 mutations/kb. We used the NGS data to assess the mutational bias in our library of >4000 hIP receptor clones (Table 5). We found no bias for the mutation of purines and pyrimidines, *i.e*. A's and T's were mutated at a similar rate as G's and C's (ratio 0.76). Transition mutations were more abundant than transversions (ratio 1.36). Mutations resulting in C's or G's were ∼4 times less likely than mutations resulting in T's and A's, favoring substitution in certain amino acids over others. These results are in accordance with previous reports and highlight the known mutational bias resulting from polymerase mediated mutagenesis [29], [32]. Comparison of the expected codon changes (considering an unbiased mutation rate and one nucleotide substitution per codon) with the observed codon changes in our NGS data showed a good overlap, indicating that the bias in nucleotide substitution only partially translates into a bias in amino acid substitutions (Figure 1H) [33]. Taken together, these data demonstrate the utility of NGS for validating the quality of mutagenesis libraries and for providing valuable information about coverage, mutation rate and potential mutational bias. Further optimization and the development of novel mutagenesis techniques are needed to generate mutagenesis libraries with less bias and increasing biological diversity.

**Table 4:** NGS results: Number of reads for each base are shown relative to reference base.

	Read_A	Read_C	Read_G	Read_T
Ref_A	2649114	244	2231	1511
Ref_C	4832	9716863	903	8154
Ref_G	7020	1206	7653501	2987
Ref_T	2869	4218	478	4624499

**Table 5:** Characteristics of the hIP receptor mutant library (4000 plasmids).

Transition (Ts)	%	Transversion (Tv)	%
A->G	55.97	A->T	37.88
T->C	55.75	T->A	38.30
G->A	60.02	A->C	6.12
C->T	58.07	T->G	6.11
		G->C	10.72
**Bias indicator**		C->G	6.51
Ts/Tv	1.37	G->T	26.63
AT->GC/GC->AT	0.75	C->A	34.33
A->N, T->N	51.39%		
G->N, C->N	48.61%	**Mutation rate**	1.48 mut/kb

### Functional characterization of hIP receptor library identified 32 mutants with reduced activity

**Figure 2:**
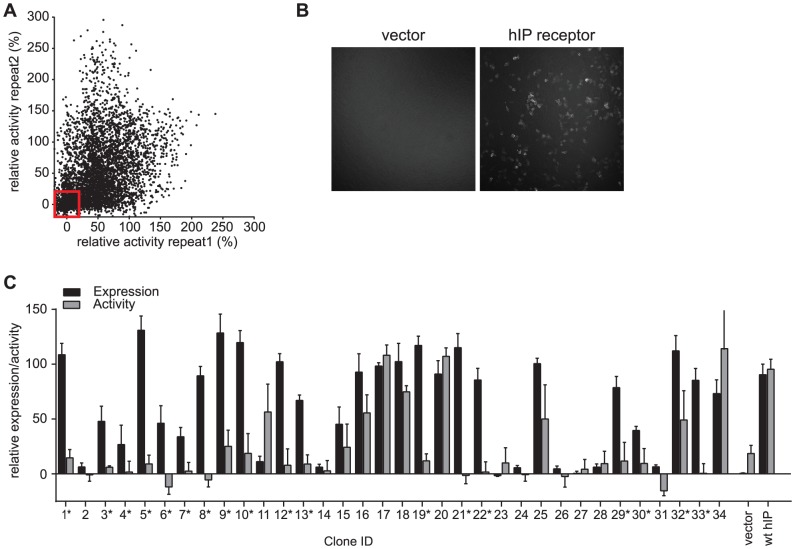
Error-prone PCR mediated mutagenesis of hIP receptor identified 32 mutants with reduced activity. (A) Scatter blot showing activity data of the hIP receptor library in the primary screen. Cells stably expressing the GloSensor cAMP plasmid were transfected with our hIP receptor constructs and stimulated with 1 µM MRE-269. The luminescent signal was normalized to the signal obtained in hIP receptor wild-type transfected cells. The experiment was performed in duplicates, shown on separate axis. Red box indicates clones with <25% activity as compared to wild-type hIP receptor. (B) Exemplary images of vector or hIP receptor expressing cells, stained with Alexa-647 anti HA-antibody. (C) Bar chart showing the relative expression level (as assessed by staining with Alexa-647 anti-HA-antibody) and the relative activity (as assessed by GloSensor cAMP assay) for 34 selected constructs. Asterisk indicates mutants classified as hits. Data were normalized to hIP receptor wild-type expressing cells and represents the mean ± SEM of three independent experiments.

In order to identify functional mutations of hIP receptor we tested the >4000 mutants in our library for activity using hIP receptor-mediated production of cAMP as a readout. The GloSensor cAMP assay utilizes a genetically encoded biosensor variant with cAMP binding domains fused to luciferase. Upon binding to cAMP, conformational changes occur that promote large increases in light output upon addition of a chemiluminescent substrate [34], [35]. We transiently transfected HEK293T cells stably expressing the GloSensor cAMP construct with our hIP receptor constructs and stimulated cAMP production with the selective hIP receptor agonist (4-[(5,6-Diphenylpyrazin-2-yl)(isopropyl)amino]butoxy)acetic acid (MRE-269) [36]. Primary hits were defined as mutants that resulted in <25% of the response to MRE-269 in cells transfected with the wild-type hIP receptor (Figure 2A). To eliminate false-negative hits due to insufficient transfection or deficiencies in trafficking of the receptor, we validated cell surface expression of ∼800 hIP receptor mutants in a subsequent step, utilizing the extracellular, N-terminal HA-tag of the protein. High content imaging of non-permeabilized cells stained with a fluorescently-labeled anti HA-antibody identified 34 mutants which demonstrated a loss of hIP receptor function but showed some cell surface expression of the receptor (for representative images see Figure 2B). These mutants were selected for analysis by sequencing, and cell surface expression and functional characterization were confirmed in 3 independent repeats of the assay (Figure 2C). Of these 34 mutants, 18 were expressed at the cell surface and demonstrated full or partial loss of receptor function.

### Random mutagenesis of hIP receptor identified inactive mutants

**Figure 3:**
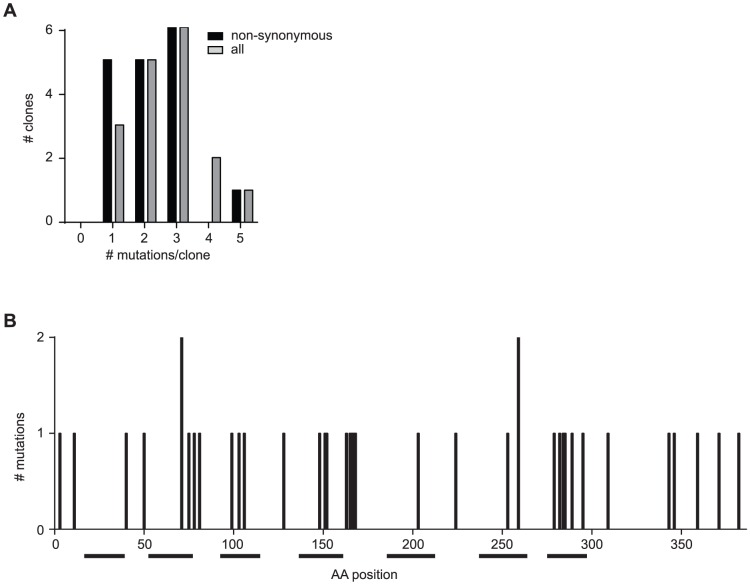
Functional characterization of hIP receptor identified inactive mutants of hIP. (A) Bar chart depicting the number and distribution of amino acid changes found in the 18 inactive mutants. Black bars represent the total number of amino acid changes; grey bars represent non-synonymous mutations only. (B) Bar chart showing the number and position of all 38 identified non-synonymous mutations in hIP receptor. Black boxes represent the α-helical, membrane-spanning domains of hIP receptor.

The observed distribution of mutations within the 18 clones showed an enrichment in clones with >1 mutations as compared to the distribution of the complete library (Figure 3A, Table 6), reflecting the higher probability of accumulated mutations to result in non-functional sequences [31]. A total of 38 non-synonymous mutations were identified within the coding region of hIP receptor, mapping to 36 distinct residues across the whole length of the protein (Figure 3B, Table 6). As depicted in Table 6 and Figure 3A, most of the mutants contained more than one non-synonymous mutation, making the interpretation challenging. Further work is therefore required to deduce which mutations are causative of loss of function. However, our hits included several previously reported mutations affecting the signaling of hIP receptor (Table 7). As shown in Figure 3B, our data identified three regions of hIP receptor with an accumulation of inactive mutations, indicating regions critical for hIP receptor function: 1) the region around Tyr-75, 2) the region around Cys-165 and 3) the region around Pro-289. Stitham et al. identified the highly conserved residues Arg-279 and Tyr-75 as lying within the PGl_2_ binding pocket of the hIP receptor and to be critical for ligand binding [14]–[16]. Cys-165 is described to form a non-conserved disulfide-bridge in human hIP receptor and mutations at this position has been shown to affect receptor expression, dimerization and activation [2], [3]. Pro-289 maps to TMVII and is part of the highly conserved DPXXF motif of the hIP receptor, a region involved in receptor activation [14], [15]. Our list of mutations also included a mutation of Asn-78 to Ile (N78I), a known glycosylation site of IP-receptor and previously implicated in receptor activation and ligand binding [37], Met-99, previously identified to contribute to ligand binding [16] and Ser-106, described to contribute to the process of signal transduction and receptor activation [17]. Notably, we identified the mutations shown in Tables 6 and 7 and Fig. 3B in a single random mutagenesis experiment. Thus, our findings corroborate the advantages of an unbiased random mutagenesis approach over targeted, site-directed mutagenesis of selected residues in multiple experiments. Taken together, these data prove the usefulness of random mutagenesis to gain insights in the structure-function requirements of hIP receptor and to identify mutations that prevent activation of the receptor.

**Table 6:** Summary of identified non-synonymous mutations in hIP-receptor.

Clone ID	mutations				
1	A40S	C259Y	F295S		
3	L81V	C151Y	C309R		
4	**C165R**	P359L			
5	G103R	**S106Y**	A382V		
6	N78I	E343D	C346R		
7	D3E	L253M	A282T		
8	**P289L**				
9	V71M				
10	**M99T**	L224P	C259R		
12	N203I	V371E			
13	V11M	G167D			
19	V71M	A128T	V148I	S168N	**R279L**
21	P285L				
22	**Y75F**				
29	A50E	P166S			
30	Q163R				
32	A152T	N284K			

Amino acid previously identified to be critical for hIP-function are in bold. Clone 33 contained a mutation in the HA-tag.

**Table 7:** Overview of identified mutations in hIP-receptor previously reported in literature.

Mutation		
identified	previously reported	Literature
R279L	R279C	[16]
P289L	P289A	[15]
Y75F	Y75A	[16]
M99T	M99L	[16]
S106Y	S106A	[17]
N78I	N78Q	[37]
C165R	C165S	[3]

## Conclusions

The recent advantages in sequencing techniques have allowed us to study genetic variants of genes at a genome-wide scale. A growing list of studies and clinical trials has started to investigate the role of SNPs in the origin and natural progression of diseases. However, the great number of identified variants and the often low prevalence have complicated the studies and only an unexpectedly small number of novel examples linking genetic variations to cardiovascular disease have been described [38]. Recent genomic sequencing studies have identified numerous SNPs in the PTGIR gene encoding the hIP receptor [14], [39]–[41]. Comparison of these mutations with the available data of previously biochemically characterized mutants has revealed a correlation between genetic variants in the hIP receptor resulting in deficits of hIP receptor function with increased disease severity in patients with coronary artery disease [10], [11]. Thus, detailed biochemical analysis of genetic variances in functional assays and insights into the structure/function relationship of the protein of interest can support the discovery of functional correlations of naturally occurring genetic variances and disease. In addition, information about the structural requirements underlying receptor function and dysfunction can aid to guide rational drug design and the development of novel therapies for cardiovascular disease.

Here we compared the applicability of chemical mutagenesis and epPCR mediated mutagenesis to generate an unbiased library of hIP receptor mutant plasmids, covering the complete coding region of PTGIR. We found that while chemical mutagenesis is especially useful to generate libraries with low frequencies of mutations it was not suitable for receptor mutation studies like the hIP receptor, where higher mutation rates are needed to guarantee sufficient coverage of the complete coding sequence [32], [42]. We describe a simple algorithm to simulate the optimal number and distribution of mutated amino acid in mutagenesis libraries as a function of the mutation rate. Using this simulation we determined the optimal mutation rate needed to generate a library of >4000 mutants of the hIP receptor by epPCR. We analyzed the quality of our library using NGS and present NGS as a rapid and non-laborious approach to determine the exact mutation rate and the mutational bias in the final library. The functional characterization of our library of >4000 mutants of the hIP receptor generated by epPCR identified 18 mutants of the hIP receptor with partial or complete loss of receptor activity while maintaining cell surface expression, translating into a hit rate less than 0.5%. Given the ∼800 mutants with impaired receptor activity in our primary screen (∼18% hit rate – not controlled for membrane expression), the difference in hit rate highlights the importance to determine the optimal mutation rate needed to avoid the accumulation of multiple mutations in one construct, thereby increasing the risk of preventing proper folding and trafficking to the membrane. The mutations identified in this mutagenesis screen included multiple novel mutations as well as mutations mapping to residues previously reported to affect the signaling of the hIP receptor [9], [11], [14]–[17], [37]. In these 18 constructs, we identified mutations across the whole length of the protein, mapping to 36 distinct residues of the hIP receptor. Due to the prevalence of multiple mutations within each construct, further work is required to characterize the novel mutations identified in this study. Notably, all these mutants were identified in a single experiment, highlighting the utility of unbiased mutagenesis approaches to investigate the structure-function relationship of GPCRs and other protein classes. Taken together, the results presented in this study demonstrate that epPCR mediated mutagenesis is a valuable method for unbiased screening of residues regulating hIP receptor expression and function.

## Supporting Information

Table S1 Worksheet to simulate the expected number and distribution of amino acids in mutagenesis libraries as a function of the mutation rate. Insert information in the yellow highlighted fields.
(XLSX)
